# Deciphering genetic factors contributing to enhanced resistance against Cercospora leaf blight in soybean (*Glycine max* L.) using GWAS analysis

**DOI:** 10.3389/fgene.2024.1377223

**Published:** 2024-05-10

**Authors:** Jinesh Patel, Tom W. Allen, Blair Buckley, Pengyin Chen, Michael Clubb, Leandro A. Mozzoni, Moldir Orazaly, Liliana Florez, David Moseley, John C. Rupe, Bishnu K. Shrestha, Paul P. Price, Brian M. Ward, Jenny Koebernick

**Affiliations:** ^1^ Department of Crop, Soil, and Environmental Sciences, Auburn University, Auburn, AL, United States; ^2^ Delta Research and Extension Center, Mississippi State University, Stoneville, MS, United States; ^3^ LSU AgCenter, Red River Research Station, Bossier City, LA, United States; ^4^ Fisher Delta Research Center, MO University of Missouri, Portageville, MO, United States; ^5^ Department of Crop, Soil, and Environmental Science, University of Arkansas, Fayetteville, AR, United States; ^6^ Department of Plant Pathology, University of Arkansas, Fayetteville, AR, United States; ^7^ LSU AgCenter, Macon Ridge Research Station, Winnsboro, LA, United States; ^8^ Department of Plant Pathology and Crop Physiology, LSU AgCenter, Baton Rouge, LA, United States

**Keywords:** GWAS, soybean, cercospora leaf blight (CLB), SNP, disease resistance

## Abstract

Cercospora leaf blight (CLB), caused by *Cercospora cf. flagellaris*, *C. kikuchii*, and *C. cf. sigesbeckiae*, is a significant soybean [*Glycine max* (L.) Merr.] disease in regions with hot and humid conditions causing yield loss in the United States and Canada. There is limited information regarding resistant soybean cultivars, and there have been marginal efforts to identify the genomic regions underlying resistance to CLB. A Genome-Wide Association Study was conducted using a diverse panel of 460 soybean accessions from maturity groups III to VII to identify the genomic regions associated to the CLB disease. These accessions were evaluated for CLB in different regions of the southeastern United States over 3 years. In total, the study identified 99 Single Nucleotide Polymorphism (SNPs) associated with the disease severity and 85 SNPs associated with disease incidence. Across multiple environments, 47 disease severity SNPs and 23 incidence SNPs were common. Candidate genes within 10 kb of these SNPs were involved in biotic and abiotic stress pathways. This information will contribute to the development of resistant soybean germplasm. Further research is warranted to study the effect of pyramiding desirable genomic regions and investigate the role of identified genes in soybean CLB resistance.

## 1 Introduction

Soybean is a major oil-producing crop grown around the world with biotic stresses restricting global production (Hartman et al., 2015). Some major yield reducing diseases in soybean include frogeye leaf spot (caused by *Cercospora sojina* Hara), Phytophthora root and stem rot (caused by *Phytophthora sansomeana* E. M. Hansen and *P. sojae* Kaufm. & Gerd.), Cercospora leaf blight [caused by *Cercospora cf. flagellaris* Ellis & G. Martin, *C. kikuchii* (Tak. Matsumoto & Tomoy) M.W. Gardner, C. *cf.* nicotianae, and *C. cf. sigesbeckiae* Katsuki], soybean cyst nematode (caused by *Heterodera glycines* Ichinohe), and charcoal rot [caused by *Macrophomina phaseolina* (Tassi) Goid.] (Wrather et al., 2010; Allen et al., 2018; Bandara et al., 2020; Bradley et al., 2021). Cercospora leaf blight (CLB) typically develops in the upper canopy, and can be observed on leaves, petioles, stems, and pods, progressing from the upper canopy downward through the plant canopy (Walters, 1980). In addition to CLB producing symptoms on all plant parts, purple seed stain (PSS) can occur on soybean kernels and is caused by the same group of organisms (Alloatti et al., 2015; Albu et al., 2016; Turner et al., 2020). Favorable environmental conditions, such as high relative humidity, prolonged dew period, and warm temperatures, influence the development of CLB (Schuh, 1992, Schuh, 1993). In general, CLB symptoms begin during reproductive stages generally during the beginning seed stage (R5) as purplish bronze-colored necrotic lesions on leaves that vary in size and elongated reddish-purple lesions on petioles (Chanda et al., 2014).

The yield losses that result from CLB are generally related to the severity of disease in the field. Numerous lesions on the leaves can result in heavy premature defoliation, delay soybean plant senescence, and reduce the production, and size of the kernels produced (Walters, 1980). Based on a survey in 2006, global yield losses taken collectively for CLB as well as purple seed stain were approximately 1,912 thousand metric tons (Wrather et al., 2010). In the U.S. at that time, CLB was considered a minor disease due to generally low yield losses. However, more recently it has become more prevalent in the southern U.S. (Cai et al., 2009; Geisler, 2013). From 2015 to 2019, CLB caused estimated yield losses of approximately 969 metric tons from 28 growing states in the U.S. and Ontario, Canada (Bradley et al., 2021).

Multiple fungal species within the genus *Cercospora* have been associated with CLB (Soares et al., 2015; Albu et al., 2016; Sautua et al., 2020b). Phylogenetic studies have previously been conducted using different molecular markers and DNA sequencing to understand the genetic diversity, variation in pathogenicity, and the presence of different lineages (Imazaki et al., 2006; Cai and Schneider, 2008; Lurá et al., 2011; Soares et al., 2015). A genetic diversity study focusing on 164 *C. kikuchii* isolates from Louisiana grouped the species in two lineages and observed that isolates in lineage II (older lineage) were more aggressive than the dominating lineage I (newer lineage) (Cai and Schneider, 2008; Cai et al., 2009). Isolates collected from Argentina, Brazil, and the U.S. were observed to contain four lineages with recombination between the second and third lineage and manifesting cryptic speciation of the pathogens associated with CLB (Soares et al., 2015). Understanding the host-pathogen interactions and ecology of these cryptic species makes defining the causal organisms of the disease difficult and adds challenges to the potential development of new monitoring tools aimed at more efficient disease management strategies (Stergiopoulos and Gordon, 2014).

General disease management strategies for CLB include crop rotation, tillage, early planting dates, the use of resistant cultivars, and the application of fungicides during reproductive stages. Fungicide applications have been a primary approach to managing CLB. However, such applications have a chance of producing fungicide-resistant strains and may have negative environmental effects (Price et al., 2015; Sautua et al., 2019; Sautua et al., 2020a). Conversely, planting disease-resistant genotypes is an alternative approach to manage CLB as they reduce fungicide applications and are cost-effective. However, at present CLB-resistant cultivars are not available and as a result soybean producers rely heavily on fungicide products that come from multiple chemical classes to reduce the yield losses associated with CLB (Carmona et al., 2011; Sautua et al., 2020a).

Research has previously been conducted to identify soybean germplasm resistant to CLB and PSS (Orth and Schuh, 1994; Alloatti et al., 2015; Li et al., 2019; Kashiwa et al., 2021; Ward et al., 2021). However, minimal research efforts have been made to identify the genes or the genomic regions that confer resistance to the pathogen causing CLB identified on the vegetative structures of the plant, but the inheritance of PSS resistance and QTLs have been identified for symptoms related to the seed (Jackson et al., 2008; Alloatti et al., 2015). Genome-Wide Association Studies (GWAS) have been used in many pathosystems to identify genomic regions and genes in the surrounding region of associated markers that can contribute to the trait of interest. GWAS leverages the power of natural diversity in germplasm and captures the historical recombinant event, thus having greater resolution than traditional linkage mapping using biparental population (Yu and Buckler, 2006). Research has been conducted to explore genomic regions that contribute to agronomically essential traits in soybean (www.soybase.org, accessed on 12/15/2023). This study reports the first GWAS study to identify genomic regions conferring resistance to CLB and putative genes that play an essential role in plant defense mechanisms. The study aims to identify genomic regions that can be used for marker-assisted selection in soybean breeding programs that focus on developing CLB resistance germplasm.

## 2 Materials and methods

### 2.1 Evaluating soybean accessions for CLB

A total of 568 soybean accessions (Supplementary Table S1) with maturity groups ranging from III to VII were obtained from the USDA germplasm collection and evaluated for CLB for 3 years (2016, 2017, and 2018) at different locations in the mid-southern U.S. Soybean accessions were planted in 21 environments: Five locations in 2016 (Fayetteville, Marianna, and Stuttgart, AR; Bossier City, LA; and Stoneville, MS) and eight locations in 2017 and 2018 (Fayetteville, Keiser, Rohwer, and Stuttgart, AR; Alexandria and Bossier City, LA; Portageville, MO; and Stoneville, MS). Planting was done during mid-June, but generally differed at each location to maximize infection and CLB symptom expression. Seed was planted in a plot represented by a single row of each accession measuring approximately 3 m in length, and the distance between each row was approximately 100 cm; however, this varied by location and ranged from 96.5 to 101.6 cm across the study locations. Recommended agronomic practices for irrigation and fertilization were conducted as well as weed and insect management.

We depend on natural inoculum because it was not practical to produce large quantities of conidia for this project. We carefully evaluated the accessions for their response to CLB during seed growth stages, especially between R5 and R6. We assessed each accession about three times during these critical growth stages. In 2016 and 2017, accessions were evaluated for CLB with different rating schemes involving observation of symptom expression from multiple specific plant parts (e.g., leaves and petioles were evaluated separately) and standard symptoms such as purpling/bronzing, blight, and petiole lesions. In 2018, a simplified severity rating scale of 0–6 to evaluate CLB severity on an additive scale based on the presentation of symptoms throughout the canopy whereby 0 for no disease symptoms, 1 for light purple/bronzing, few petiole lesions, no leaf blight, 2 for moderate purple/bronzing and/or petiole lesions, no or minimal leaf blight, 3 for heavy purple/bronzing and/or petiole lesions, light blight, 4 for heavy purple/bronzing and/or petiole lesions, moderate blight, 5 for severe blight and less than 50% defoliation, and 6 for severe blight but more than 50% defoliation. In addition, percent incidence was presented on a 0 to 4 scale to encompass observational incidence as quartiles (Ward et al., 2021). Natural infection was relied on, and several locations with low disease pressure were not used in the final GWAS analysis. Locations with moderate to heavy disease pressure were used for GWAS analysis and included: Bossier City, LA (BLA) and Stoneville, MS (SMS) in 2016; Alexandria, LA (ALA), Bossier City, LA, and Stoneville, MS in 2017; and Alexandria and Bossier City, LA, Stoneville, MS, and Fayetteville, AR (FAR) in 2018.

### 2.2 Accession and SNP array data for GWAS

As a result of seed scarcity there was variation in the number of accessions planted at each location and genotypic data for all 568 accessions was not available. Therefore, 460 accessions that were planted and evaluated in all the location-years as well as with available genotypic data were used for analyses. A total of 42,080 SNPs that can be assigned to 20 chromosomes within the soybean genome were obtained through the Illumina Infinium SoySNP50 K iSelect SNP Beadchip (Song et al., 2013). Markers with minor allele frequency (MAF) < 5% or had a missing rate greater than 10% were excluded, resulting in 31,491 SNPs for additional analysis.

### 2.3 Population structure and linkage disequilibrium analysis

Population structure analysis to determine the number of subpopulation and member of each subpopulation was done using STRUCTURE v.2.3.4 software (Pritchard et al., 2000). For structure analysis, the resulting 31,491 SNPs were used with admixture model with five iterations of 50,000 burn-in and 50,000 Monte Carlo Markov Chain (MCMC) replications for k = 1 to k = 10. The number contained within the subpopulation was determined by calculating DeltaK using Structure Harvester (Earl and Vonholdt, 2012). Furthermore, linkage disequilibrium (LD) decay or r2 between pairs of markers was calculated using TASSEL (Bradbury et al., 2007) using sliding windows of 50 SNPs, and visualization was done in R to determine the LD decay rate and distance when the r2 dropped to half of the maximum value (Remington et al., 2001). Linkage disequilibrium between pairs of markers was plotted against the physical distance relying on the *r* ^2^ values. The red fitting curve estimates the rate of LD decay when the power of maximum *r* ^2^ was reduced to half, and the blue fitting curve illustrates the model fit to LD decay.

### 2.4 Assessing phenotypes and statistical analysis

The combined data observations for the years 2016 and 2017 were converted into categorical data and analyzed using a linear model with the “lm” function. The “anova” function was then used to obtain an ANOVA table. For the 2018 data, ANOVA was performed using the “aov” function, considering the severity and incidence. ANOVA was calculated separately for the years 2016-17 and 2018, as different methods and scales were used to rate CLB disease.

The program TASSEL was used to remove markers with minor allele frequency (MAF) < 5% or with a missing rate larger than 10% (Bradbury et al., 2007). The filtered set of markers were used to conduct GWAS using FarmCPU (Fixed and random model Circulating Probability Unification) with one of the tools present in GAPIT version 3 package in R (Liu et al., 2016; Wang and Zhang, 2021). FarmCPU is more effective than the generalized linear model (GLM) and the mixed linear model (MLM) as it controlled with both false positives and false negatives and is a widely used method in soybean GWAS studies (Kaler et al., 2018; Chamarthi et al., 2021). Kinship and PCA analysis were conducted using GAPIT and incorporated in the GWAS analysis. A SNP was declared significantly associated with the trait if it crossed the threshold value, [−Log10 (P) ≥ 3.5] or had a *p*-value ≤ 0.0003, which is similar to previous soybean GWAS studies (Dhanapal et al., 2015; Kaler et al., 2018; Chamarthi et al., 2021). If a SNP had a significant association to a trait [−Log10 (P) ≥ 3.5] in at least one environment and passed a threshold value of *p* ≤ 0.05 in additional environments, it was considered to be a common significant associated SNP for multiple environments (Kaler et al., 2017; Kaler et al., 2018; Chamarthi et al., 2021).

Candidate genes were searched for SNPs that were associated with CLB in multiple environments by scanning a ∼20 kb region of the significant SNP. The genome browser at Phytozome (https://phytozome.jgi.doe.gov/jbrowse/) and SoyBase (https://soybase.org) was used to identify putative genes and their functions. The search was targeted to identify any genes that are involved in defense mechanisms against biotic and abiotic stresses.

## 3 Results

### 3.1 Assessing CLB disease in soybean accessions

The ANOVA analysis indicated that in 2016 and 2017, the accessions significantly impacted CLB severity more than the locations. In 2018, the locations became the primary cause of variation in plant response to CLB, with the accessions contributing a lesser role (Supplementary Table S3, adapted from Ward et al., 2021). All accessions planted over the 3 years were ranked from most resistant to least resistant based on normalized disease evaluations (Supplementary Table S4, adapted from Ward et al., 2021). Among the 460 accessions evaluated, 17 consistently showed resistance to CLB for all 3 years and appeared in the top 10% of the population with the lowest disease severity (Supplementary Figure S1; Supplementary Table S4, adapted from Ward et al., 2021). Similarly, 17 accessions were identified consistently higher level of the disease severity across the years. These accessions will provide excellent genetic resources for cloning the genes that interact with the pathogen and understanding the interaction between the host and the pathogen.

### 3.2 Genomic insights: understanding marker distribution, LD decay, and population structure

The filtered SNPs amounted to 31,491 in total, spanning an estimated 948 megabases (Mb), which covers approximately 82% of the soybean genome (1.15 Gb). Across the chromosomes, the number of SNP markers per chromosome exhibited a range from 998 (chromosome 20) to 2,694 (chromosome 18), with an average of 1,575 SNP markers per chromosome (Table 1). The average distance between the two markers for the genome was approximately 30.1 Kb, while the range varied from 21.5 Kb (chromosome 18) to 48 Kb (chromosome 20).

**TABLE 1:** Distribution of SNP markers along the soybean genome.

Total number of base pairs (bp)			
Chr^a^	Total SNP	Chr length	Avg. SNP distance^b^
1	1,256	56,830,220	45,247
2	1,875	48,567,990	25,903
3	1,274	45,712,413	35,881
4	1,454	52,360,037	36,011
5	1,381	42,090,709	30,478
6	1,517	51,316,639	33,828
7	1,625	44,608,799	27,452
8	1,869	47,796,376	25,573
9	1,468	50,149,215	34,162
10	1,572	51,501,283	32,762
11	1,156	34,725,337	30,039
12	1,076	40,077,424	37,247
13	2,101	45,487,373	21,650
14	1,570	48,997,963	31,209
15	1,982	51,670,112	26,070
16	1,376	37,828,927	27,492
17	1,534	41,600,558	27,119
18	2,694	57,968,596	21,518
19	1,713	50,730,824	29,615
20	998	47,895,551	47,992

^a^ Chromosome.

^b^ Average distance between SNPs.

**FIGURE 1:**
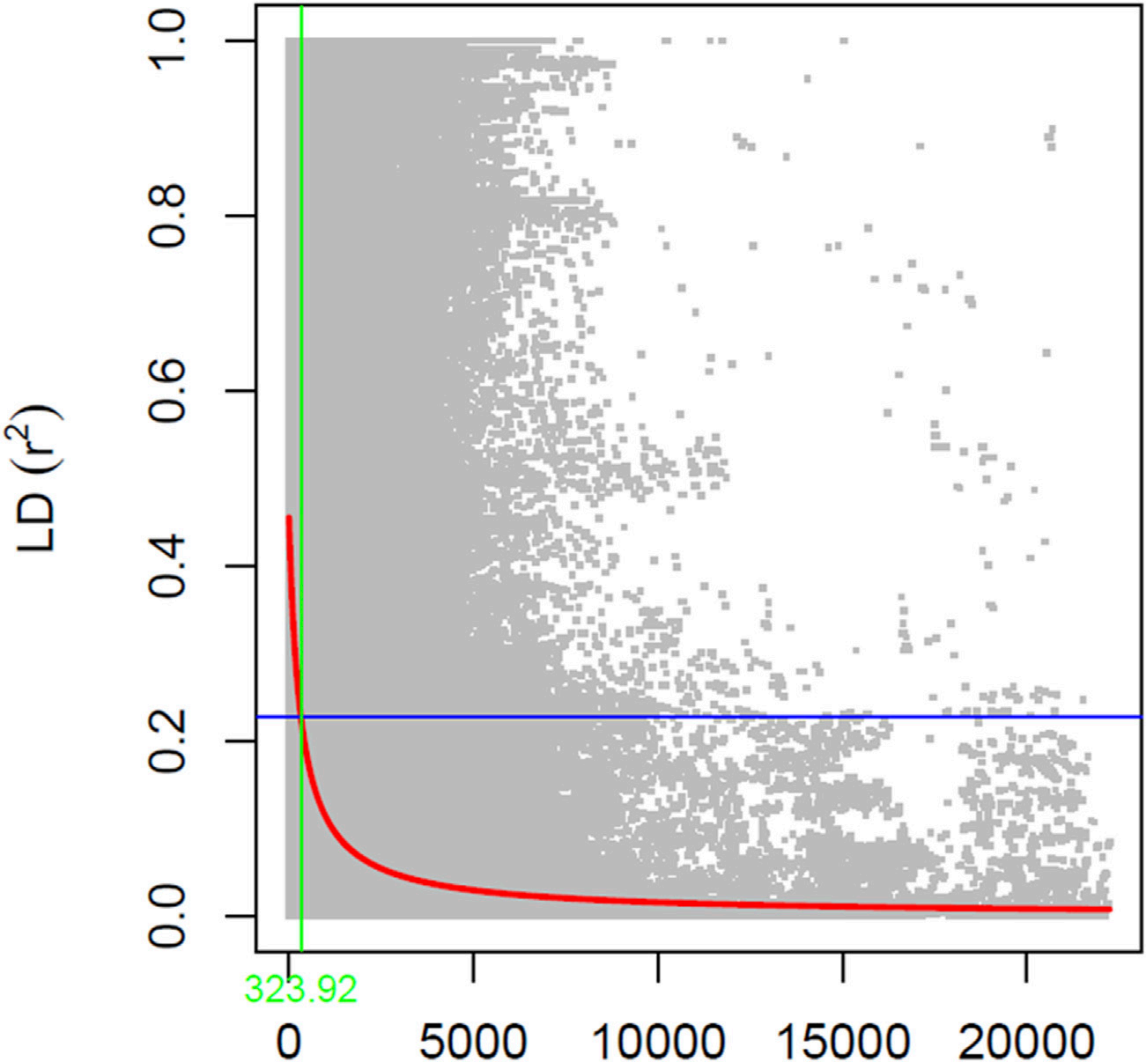
Linkage disequilibrium (LD) patterns on the soybean genome developed using 31,491 SNPs (MAF ≥0.05). The *y*-axis represents the average *r* ^2^ value, while the *x*-axis represents physical distance.

**FIGURE 2:**
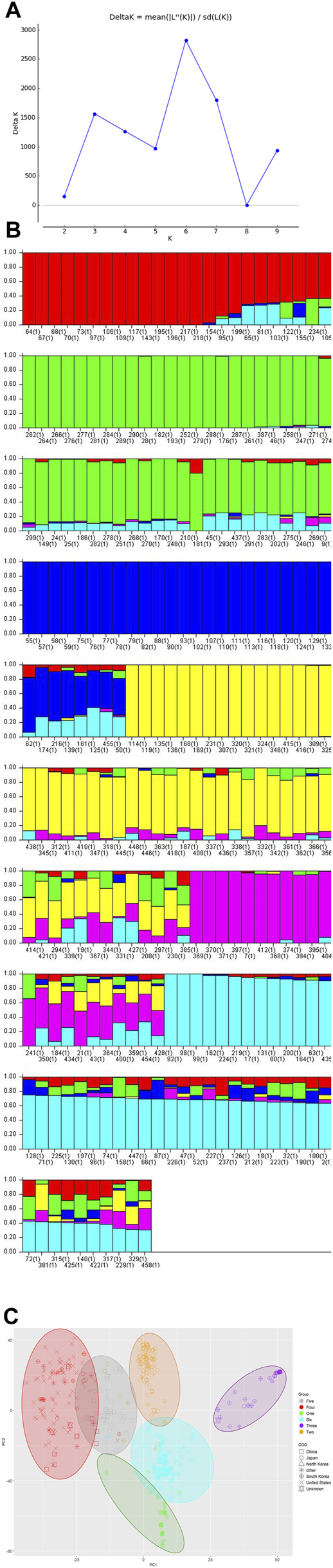
(Continued).

The estimated LD decay rate, measured in the r2, dropped to half at approximately 324 Kb (Figure 1). According to population structure evaluation, optimum subpopulation (k) was determined to be 6, which means that the 460 genotypes can be divided into six populations (Figure 2). The average distance (expected heterozygosity) within a subpopulation was approximately 0.2 while the FST fixation index, which measures the genetic variance in the subpopulation, ranged from 0.34 to 0.98 (Supplementary Table S2). Principal component analysis (PCA) clustering also supported that the accessions group into six different subpopulation (Figure 2).

### 3.3 Identification of genomic regions linked to CLB

**FIGURE 3:**
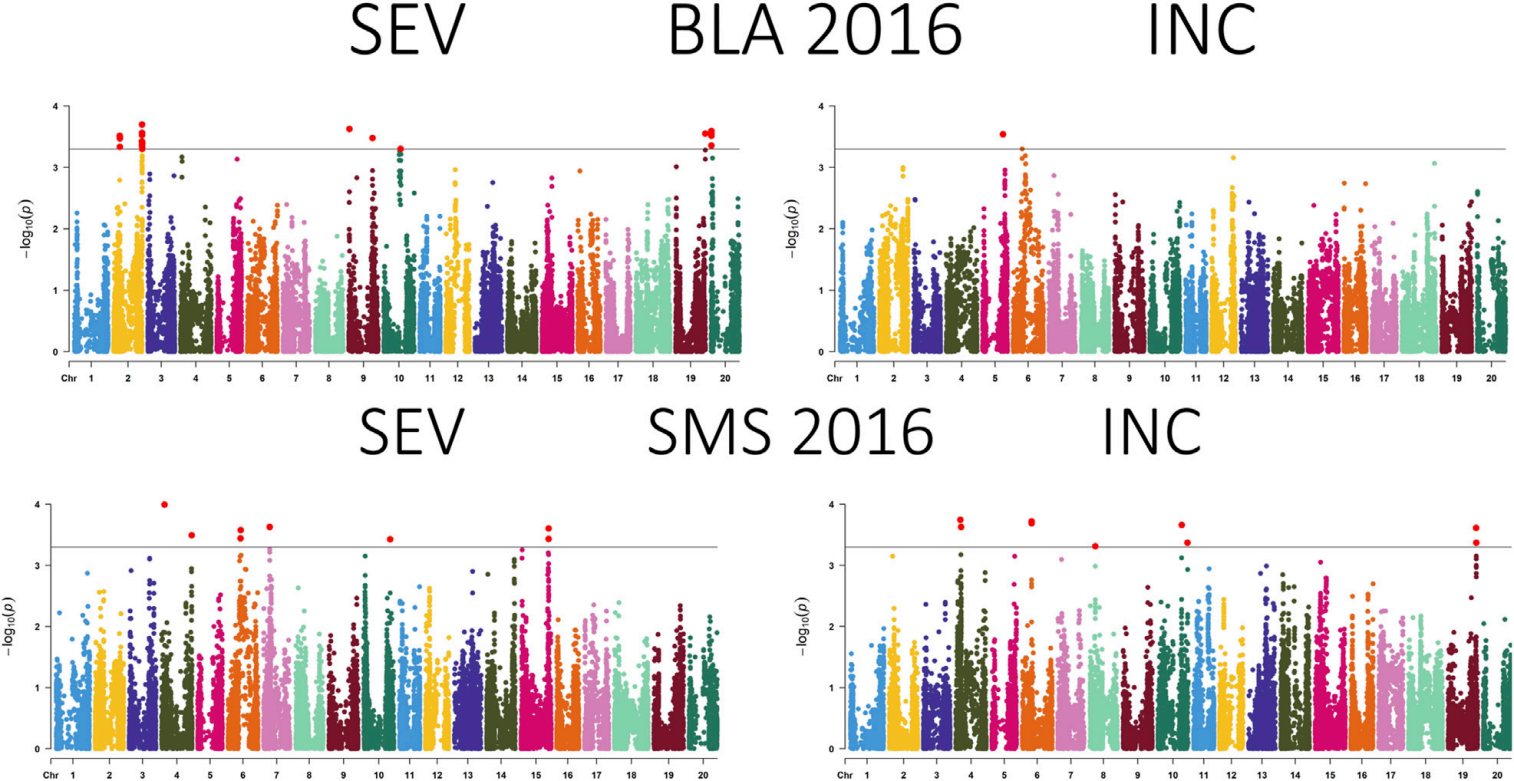
Manhattan plot presenting the association between SNPs, disease severity (SEV), and incidence (INC) of soybean accessions evaluated for Cercospora leaf blight in Bossier City, LA (BLA) and Stoneville, MS (SMS) during 2016. The gray horizontal line indicates the genome-wide significant threshold [−log10 (*p*-value) = 3.5].

**FIGURE 4:**
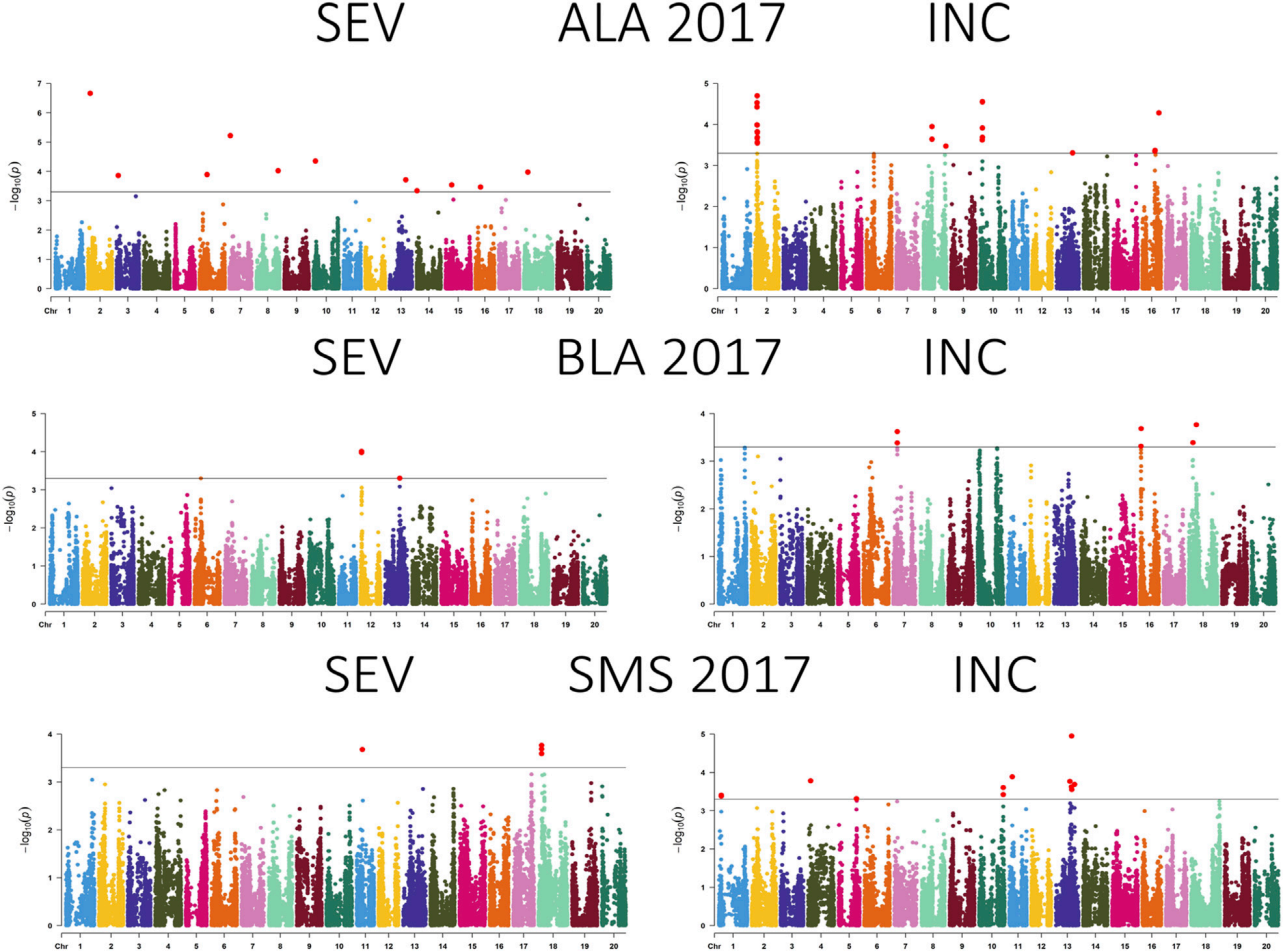
Manhattan plot showing the association between SNPs, disease severity (SEV), and incidence (INC) of soybean accessions evaluated for Cercospora leaf blight in Alexandria, LA (ALA), Bossier City, LA (BLA), and Stoneville, MS (SMS) during 2017. The gray horizontal line indicates the genome-wide significant threshold [−log10 (*p*-value) = 3.5].

**FIGURE 5:**
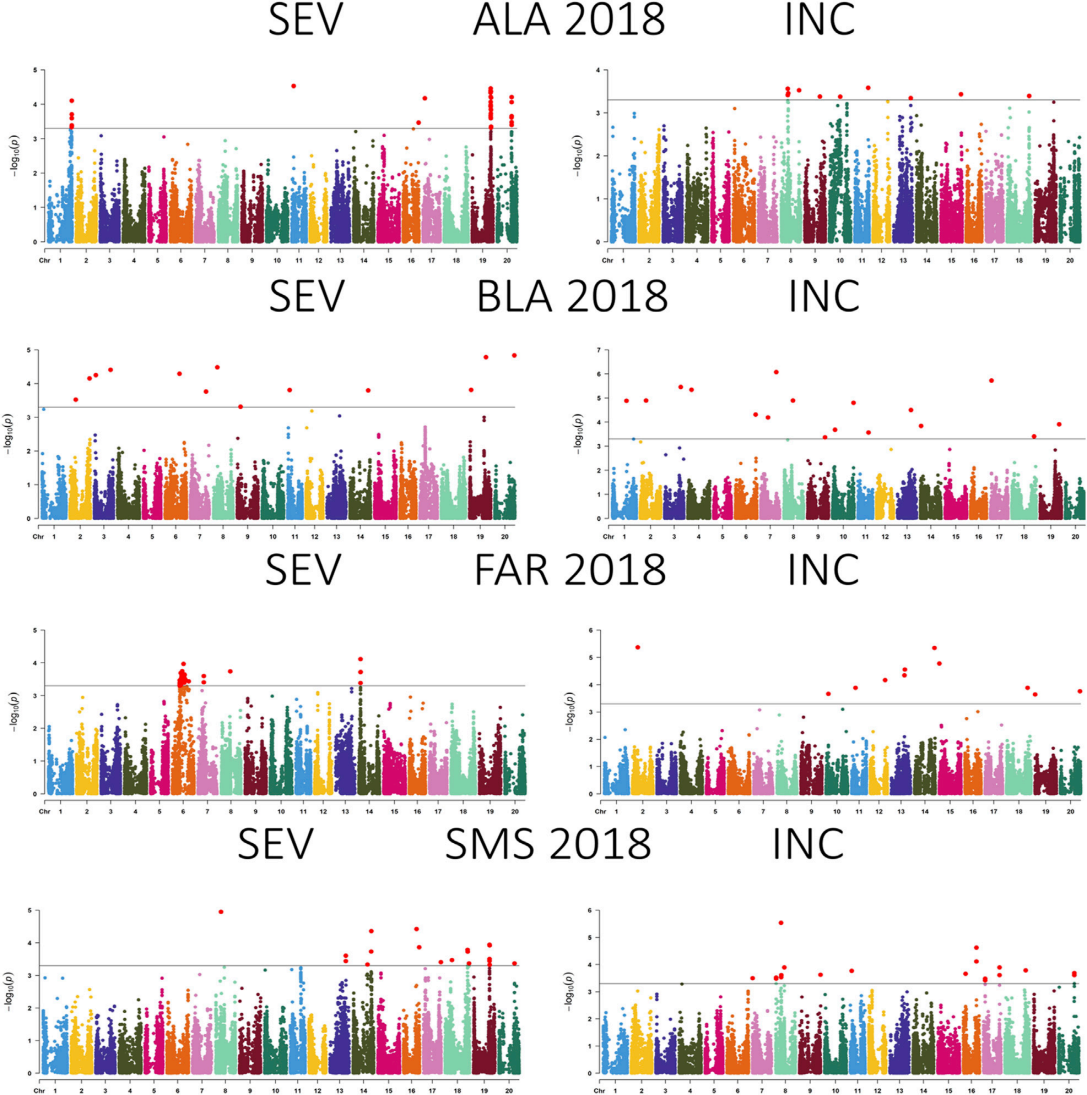
Manhattan plot showing the association between SNPs, disease severity (SEV), and incidence (INC) of soybean accessions evaluated for Cercospora leaf blight in Alexandria, LA (ALA), Bossier City, LA (BLA), Fayetteville, AR (FAR), and Stoneville, MS (SMS) during 2018. The gray horizontal line indicates the genome-wide significant threshold [−log10 (*p*-value) = 3.5].

A total of 99 SNPs in 18 of 20 chromosomes were identified to be associated with CLB severity, while 47 SNPs that were present in more than two environments were distributed in 13 chromosomes (Figures 3–5; Supplementary Figures S1–S3; Supplementary Table S5). Chromosome 19 had the greatest number of associated SNPs to severity, while chromosome 6 had the greatest number of common SNPs (Table 2). Furthermore, the allele effect for disease severity ranged from −8.68 to 9.18 for these significantly associated SNPs. Here a negative value for allelic effect suggested that minor alleles were favorable, while a positive allele effect suggested that major alleles were favorable for disease resistance. For disease incidence we observed a total of 85 SNPs associated in all 20 chromosomes with chromosome 2 having the greatest number of SNPs (Figures 3–5; Supplementary Figures S2–S4; Supplementary Table S6). A total of 23 SNPs were determined to be associated to disease incidence in more than two environments (Table 3). The allele effect for disease incidence ranged from −5.96 to 10.98 for these significantly associated SNPs.

**TABLE 2:** SNPs associated with CLB severity identified by a FarmCPU model [threshold: −Log10 (P) ≥ 3.5].

Env^a^	SNP	Chr^b^	Position (bp)	−Log10 (P)	Common env^c^	Maf^d^	Allelic effect	SNP allele^e^
ALA_2017	ss715620460	15	12632475	3.54	FAR_2018, SMS_2018, SMS_2016	0.34	1.04	**A**/G
ALA_2018	ss715635359	19	44615812	4.06	SMS_2017	0.18	−0.29	G/**T**
ALA_2018	ss715635356	19	44605399	3.98	SMS_2017	0.19	−0.28	C/**T**
ALA_2018	ss715635357	19	44608641	3.96	SMS_2017	0.18	0.28	**T**/G
ALA_2018	ss715635361	19	44666185	3.93	SMS_2017	0.18	−0.27	A/**G**
ALA_2018	ss715635400	19	44937972	3.85	SMS_2017	0.2	−0.26	G/**T**
ALA_2018	ss715635408	19	45042351	3.73	FAR_2018	0.13	−0.28	C/**T**
ALA_2018	ss715635370	19	44734953	3.67	SMS_2017	0.17	0.28	**G**/A
ALA_2018	ss715635362	19	44669507	3.62	SMS_2017	0.18	−0.26	C/**T**
ALA_2018	ss715580619	1	55775590	3.59	SMS_2017	0.27	−0.21	G/**A**
BLA_2016	ss715583504	2	48122718	3.7	FAR_2018	0.1	8.07	**A**/G
BLA_2016	ss715583501	2	48101476	3.56	FAR_2018	0.1	−7.83	G/**A**
BLA_2016	ss715583502	2	48101923	3.56	FAR_2018	0.1	7.83	**C**/T
BLA_2016	ss715583498	2	48051672	3.53	FAR_2018, BLA_2017	0.11	−7.92	T/**C**
BLA_2018	ss715638765	20	46556257	4.84	BLA_2016	0.06	−0.18	A/**G**
BLA_2018	ss715585954	3	38651529	4.41	FAR_2018	0.17	−0.11	G/**A**
BLA_2018	ss715594189	6	34566459	4.29	FAR_2018	0.06	−0.21	A/**G**
BLA_2018	ss715618917	14	43604766	3.8	SMS_2018, FAR_2018	0.27	−0.07	A/**G**
BLA_2018	ss715597528	7	37054104	3.76	BLA_2016	0.33	−0.1	T/**G**
FAR_2018	ss715618635	14	3949935	4.11	SMS_2018	0.21	0.21	**G**/A
FAR_2018	ss715594036	6	26380083	3.97	BLA_2018, BLA_2016	0.08	0.33	**A**/G
FAR_2018	ss715593943	6	23333717	3.75	BLA_2016	0.08	−0.33	C/**A**
FAR_2018	ss715618632	14	3946031	3.72	SMS_2018	0.2	−0.2	G/**T**
FAR_2018	ss715593832	6	19858251	3.69	BLA_2017	0.08	−0.32	G/**A**
FAR_2018	ss715594047	6	28403264	3.65	BLA_2016	0.07	0.34	**G**/T
FAR_2018	ss715594062	6	28978753	3.64	BLA_2016	0.08	0.32	**C**/T
FAR_2018	ss715593925	6	22783857	3.62	BLA_2016	0.08	0.32	**C**/T
FAR_2018	ss715593901	6	22036914	3.62	BLA_2016, BLA_2018	0.08	0.32	**C**/T
FAR_2018	ss715593955	6	23769222	3.59	BLA_2016	0.08	0.32	**C**/T
FAR_2018	ss715593896	6	21820219	3.56	BLA_2016	0.08	0.31	**C**/T
FAR_2018	ss715593900	6	21986774	3.56	BLA_2016	0.08	−0.31	G/**A**
FAR_2018	ss715593967	6	26660217	3.55	BLA_2016	0.08	0.32	**C**/T
FAR_2018	ss715593987	6	27526124	3.54	BLA_2016	0.08	0.32	**A**/G
FAR_2018	ss715593998	6	24802794	3.54	BLA_2016	0.08	0.32	**A**/G
FAR_2018	ss715594042	6	28131325	3.54	BLA_2016	0.08	0.32	**A**/G
FAR_2018	ss715594057	6	28804685	3.54	BLA_2016	0.08	0.32	**A**/G
FAR_2018	ss715593975	6	27937415	3.54	BLA_2016	0.08	−0.32	G/**A**
FAR_2018	ss715594050	6	28553320	3.54	BLA_2016	0.08	−0.32	T/**C**
FAR_2018	ss715594002	6	24628613	3.53	BLA_2016	0.08	0.32	**C**/T
SMS_2016	ss715608754	11	10457269	3.69	SMS_2018, FAR_2018	0.19	−4.93	C/**T**
SMS_2016	ss715600633	8	21178053	3.53	SMS_2018	0.16	−4.97	C/**T**
SMS_2017	ss715632179	18	5998461	3.77	SMS_2016	0.16	−8.68	G/**A**
SMS_2017	ss715632113	18	5961788	3.69	SMS_2016	0.16	8.61	**C**/T
SMS_2017	ss715632129	18	5971300	3.59	SMS_2016	0.16	−8.33	T/**G**
SMS_2018	ss715624926	16	36995747	3.86	SMS_2016	0.28	−0.27	G/**A**
SMS_2018	ss715631877	18	53264912	3.79	FAR_2018, BLA_2017	0.12	−0.41	C/**A**
SMS_2018	ss715631897	18	53424779	3.73	ALA_2018, FAR_2018, BLA_2017, SMS_2017	0.13	−0.39	T/**C**

^a^ Environment.

^b^ Chromosome.

^c^ Common environment.

^d^ Minor allele frequency.

^e^ Bold allele is favorable for disease resistance.

**TABLE 3:** SNPs associated with disease incidence identified by a FarmCPU model [threshold: −Log10 (P) ≥ 3.5].

Env^a^	SNP	Chr^b^	Position (bp)	−Log10 (P)	Common env^c^	Maf^d^	Allelic effect	SNP allele^e^
BLA_2018	ss715594889	6	48444236	4.31	ALA_2017	0.4	0.1	**T**/G
BLA_2018	ss715609189	11	24796245	3.57	ALA_2017	0.31	0.12	**C**/T
FAR_2018	ss715638723	20	46275367	3.76	ALA_2017	0.07	0.19	**C**/T
ALA_2017	ss715600079	8	18453233	3.64	ALA_2018	0.12	3.99	**A**/G
ALA_2017	ss715600081	8	18482110	3.95	ALA_2018	0.11	−4.27	T/**C**
FAR_2018	ss715611266	11	8860317	3.88	ALA_2018	0.23	−0.11	G/**A**
SMS_2018	ss715599461	8	13496708	5.53	BLA_2017	0.12	0.35	**G**/A
SMS_2017	ss715615115	13	31244954	3.62	BLA_2017	0.26	0.91	**G**/A
BLA_2018	ss715617757	14	1416586	3.84	BLA_2017, BLA_2016	0.08	0.18	**G**/A
ALA_2017	ss715582881	2	4603309	4.7	BLA_2018	0.06	5.94	**C**/T
SMS_2017	ss715615248	13	32098916	3.55	BLA_2018	0.26	−0.92	C/**T**
BLA_2017	ss715629154	18	15404893	3.76	BLA_2018	0.05	−1.97	C/**A**
FAR_2018	ss715615072	13	31005416	4.35	SMS_2016	0.24	−0.13	T/**C**
FAR_2018	ss715619376	14	47205840	5.35	SMS_2016	0.21	0.13	**G**/A
SMS_2016	ss715589059	4	5241170	3.99	SMS_2017	0.06	10.75	**G**/A
FAR_2018	ss715615214	13	31878146	4.56	SMS_2017	0.14	−0.13	G/**A**
FAR_2018	ss715631538	18	49825477	3.88	SMS_2017, ALA_2017	0.11	0.13	**G**/A
ALA_2017	ss715606734	10	3899657	4.56	SMS_2018	0.2	3.74	**A**/G
ALA_2017	ss715606744	10	3901046	4.55	SMS_2018	0.21	−3.68	T/**C**
ALA_2017	ss715606797	10	3913977	3.92	SMS_2018	0.18	3.59	**A**/G
ALA_2017	ss715606802	10	3916161	3.69	SMS_2018	0.19	3.45	**G**/T
BLA_2018	ss715608347	10	5954818	3.68	SMS_2018	0.12	−0.16	T/**G**
SMS_2017	ss715607846	10	48598316	3.6	SMS_2018, BLA_2016	0.18	1.04	**A**/G

^a^ Environment.

^b^ Chromosome.

^c^ Common environment.

^d^ Minor allele frequency.

^e^ Bold allele is favorable for disease resistance.

### 3.4 Candidate genes and ontologies for CLB resistance

A total of 47 and 23 SNPs (Tables 2, 3) showed a significant association and were observed to be associated in multiple environments for disease severity and incidence rating, respectively. The search for candidate genes revealed that 36 out of 47 associated SNPs for disease severity and 19 out of 23 SNPs for disease incidence had genes in the vicinity of the 10 kb region surrounding them. A total of 72 and 57 genes (Supplementary Table S7), respectively, were identified in the vicinity of 36 (disease severity) and 19 (disease incidence) SNPs. GO enrichment analysis (https://www.soybase.org/) of these genes indicate involvement in several biotic and abiotic stress pathways such as regulating hydrogen peroxide; salicylic acid metabolic process; abscisic acid mediated signaling pathway; defense response to pathogens and insects; flavonoid biosynthetic pathway; response to cold and salt stress; as well as wound response signaling.

## 4 Discussion

To date, a limited number of studies have focused on identifying genomic regions associated with resistance to *Cercospora kikuchii* causal pathogen of CLB. The QTL resistance of *C. kikuchii* resistance for PSS was identified in a F_2_ population with parents, “Agripro 350” and “PI 80837” (Jackson et al., 2008); however, this provided limited information due to the lack of recombination events and genomic composition in a single cross. In GWAS studies, natural variation among a large set of soybean germplasm is used providing greater resolution and increasing the chances of identifying additional genomic regions associated with the trait. This approach has previously been used in soybean to discover genomic regions related to essential attributes like canopy cover, lodging, leaflet area, oil and protein content, plant height, pod shattering, pod number, oil component, protein components, and abiotic and biotic stresses (Leamy et al., 2017; Kaler et al., 2018; Lee et al., 2019; Patel et al., 2023b).

A total of 99 and 85 SNPs were identified associated with disease severity and incidence, respectively, out of which 47 and 23 were confirmed over multiple environments. In total, 14 SNPs were less than 200 kb away from the QTLs that have been identified to bestow resistance to diseases such as soybean cyst nematode (SCN), sudden death syndrome (SDS), Soybean mosaic virus (SMV), and Sclerotinia stem rot (Table 4).

**TABLE 4:** Soybean genome region associated for multiple disease resistance.

Location	Year	Rating	SNP	Chr^a^	Basepair (bp)	SNP allele^b^	QTL^c^	Distance in bp
BLA	2018	CAT	ss715585954	Gm03	38,651,529	G/**A**	SCN 1-g1	171,570
BLA	2018	CAT	ss715597528	Gm07	37,054,104	T/**G**	SDS 1-g28	19,610
FAR	2018	CAT	ss715618632	Gm14	3,946,031	G/**T**	SCN 3-g11	92,359
FAR	2018	CAT	ss715618635	Gm14	3,949,935	**G**/A	SCN 3-g11	96,263
SMS	2017	CAT	ss715632113	Gm18	5,961,788	**C**/T	SCN 2-g1	147,116
SMS	2017	CAT	ss715632129	Gm18	5,971,300	T/**G**	SCN 2-g1	156,628
SMS	2017	CAT	ss715632179	Gm18	5,998,461	G/**A**	SCN 2-g1	183,789
BLA	2018	CAT	ss715638765	Gm20	46,556,257	A/**G**	SCN 5-g53	122,505
ALA	2017	INC	ss715600079	Gm08	18,453,233	**A**/G	SDS 1-g40	48,433
ALA	2017	INC	ss715600081	Gm08	18,482,110	T/**C**	SDS 1-g40	77,310
BLA	2018	INC	ss715608347	Gm10	5,954,818	T/**G**	Sclero 3-g40	50,989
FAR	2018	INC	ss715611266	Gm11	8,860,317	G/**A**	SCN 5-g25	38,561
FAR	2018	INC	ss715619376	Gm14	47,205,840	**G**/A	SMV 1-g2	175,447
FAR	2018	INC	ss715631538	Gm18	49,825,477	**G**/A	SCN 2-g8 & Sclero 3-g36	9,268 to 166,249

^a^ Chromosome.

^b^ Bold allele is favorable for disease resistance.

^c^ Quantitative trait loci.

A SNP (ss715597528) on chromosome 7 was 19 kb away from the QTL (SDS 1-g28) associated with resistance to SDS (Bao et al., 2015). Additionally, within the 10 kb region surrounding ss715597528, two leucine-rich repeat (LRR) receptor-like kinases, namely, Glyma.07g201600 and Glyma.07g201700, were identified (Supplementary Table S4). Several reports have suggested that these genes play crucial roles in providing resistance to multiple diseases (Song et al., 2008; Hok et al., 2011; Pradhan et al., 2018; Thapa et al., 2018). Leucine-rich repeat (LRR) receptor-like kinase genes represent a super family of transmembrane receptor-like kinases that are mainly involved in responding to external biotic and abiotic stress (Dufayard et al., 2017).

A SNP (ss715631538) located on Chr.18 associated with CLB was also detected in the vicinity of QTL SCN 2-g8, and QTL Sclero 3-g36, which provide SCN and SSR resistance, respectively (Zhang et al., 2016; Moellers et al., 2017). Glyma.18g211100 (peroxidase superfamily protein) and Glyma.18g210300 (guanine nucleotide-binding protein) were discovered in the surrounding 220 kb region of the SNP (ss715631538). Peroxidase superfamily protein and guanine nucleotide-binding protein both play roles in regulating stress, hormone biosynthesis, ROS metabolism, signaling pathways, and providing defense against diseases (Assmann, 2005; Brenya et al., 2016; Pandey et al., 2017; Jin et al., 2019). In addition, three SNPs (ss715632113, ss715632129, and ss715632179) distributed in the 35 kb region of chromosome 18 were determined to be associated with CLB and the QTL SCN 2-g1 was discovered less than 150 kb away (Table 4) (Zhang et al., 2016). Genes like leucine-rich repeat receptor-like protein kinase (Glyma.18g064100), RING/U-box superfamily protein (Glyma.18g063500), and ATP-binding cassette transporter (Glyma.18g063400) were found in close proximity of these three SNPs. These genes have been determined to be extensively involved in plant stress, disease response, and defense mechanisms in plants (Campbell et al., 2003; Rea, 2007; Sharma et al., 2013).

Recently, GWAS and RNA-Seq studies were conducted to identify genomic regions and genes linked to the soybean response to target spot, caused by *Corynespora cassiicola* (Patel et al., 2023a; Patel et al., 2023b). Two associated SNPs from the current study were located within 1 Mb from markers associated with target spot symptoms in soybean: One nestled on chromosome 11 (ss715608754), which demonstrated an association with the incidence of CLB and another residing on chromosome 16 (ss715624926), which was linked to the severity of CLB. The SNP ss715624926 on chromosome 16 is within a 1.86 Mb genomic region, which hosts markers that were linked not only to target spot but also to Sclerotinia stem rot, iron deficiency chlorosis, and SCN. This 1.86 Mb genomic region is home to 35 genes that are crucial in disease resistance including: Family protein/LRR family protein, TIR-NBS-LRR family, receptor-like proteins (RLP), cysteine-rich RLK protein kinase 25, cytochrome P450, LRR-RLK, and LRR transmembrane protein kinase (Bradbury et al., 2007). Further exploration of this region may open exciting possibilities for the cultivation of soybean cultivars with broad-spectrum resistance against multiple pathogens, thereby fortifying the sustainability and resilience of soybean.

## 5 Conclusion

In summary, this study marks a pioneering effort in identifying genomic regions and genes influencing soybean plant response to CLB. The 17 lines identified in this effort coupled with the identification of key SNPs and candidate genes offer considerable value for developing novel breeding lines fortified with CLB resistance. Moreover, certain genomic regions revealed in our study consistently demonstrated associations with CLB across diverse environments and have previously been linked to additional diseases. These findings present an opportunity to leverage these genomic regions, facilitating the streamlined integration of multiple disease-resistance loci in future breeding programs. To better understand our findings, additional research in controlled environments using specific species of *Cercospora* is necessary.

## Data Availability

The datasets presented in this study can be found in online repositories. The names of the repository/repositories and accession number(s) can be found in the article/Supplementary Materials.
