# Supplementary material for: Deciphering genetic factors contributing to enhanced resistance against Cercospora leaf blight in soybean (Glycine max L.) using GWAS analysis
Source: Front Genet. 2024 May 10;15:1377223. doi: 10.3389/fgene.2024.1377223 (PMC11116733; doi:10.3389/fgene.2024.1377223)
Supplement: Supplementary file 1 [file Table2.DOCX]

**
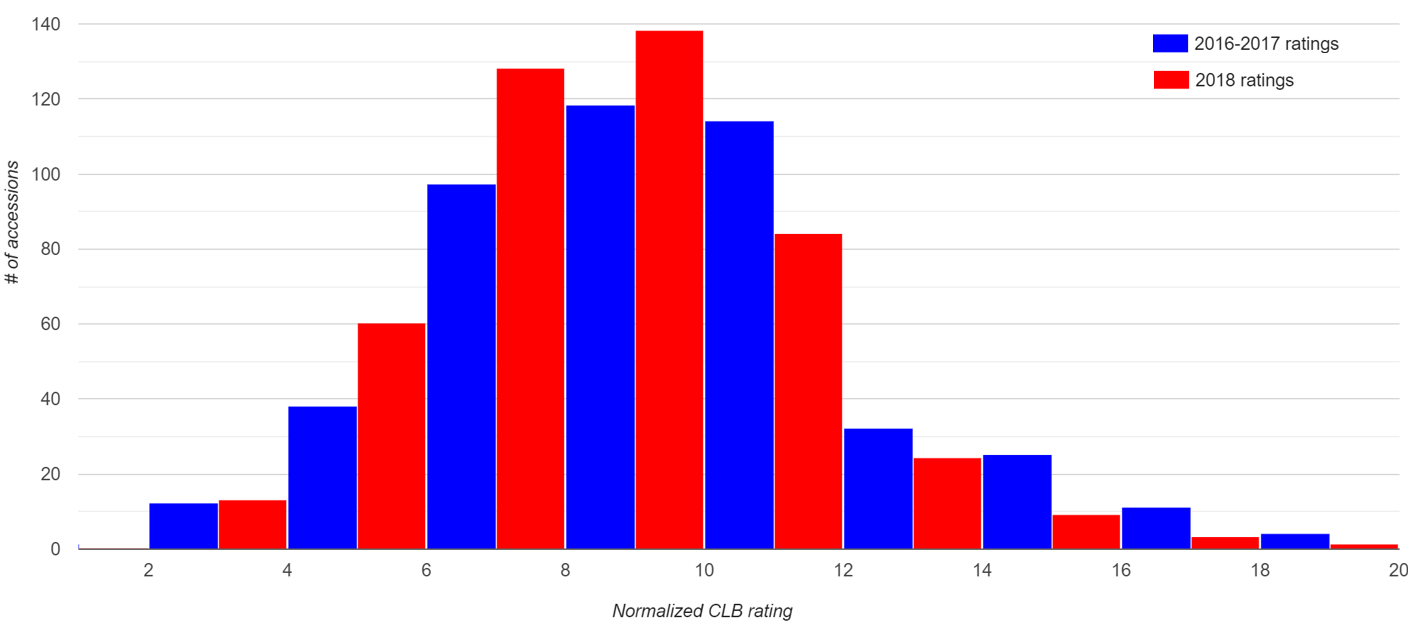
**

**Supplementary Figure 1** Histogram representing frequency distribution of CLB disease rating averages for the 2016/17 and 2018 data sets. The Y-axis represents disease rating, and the X-axis represents disease rating.

| 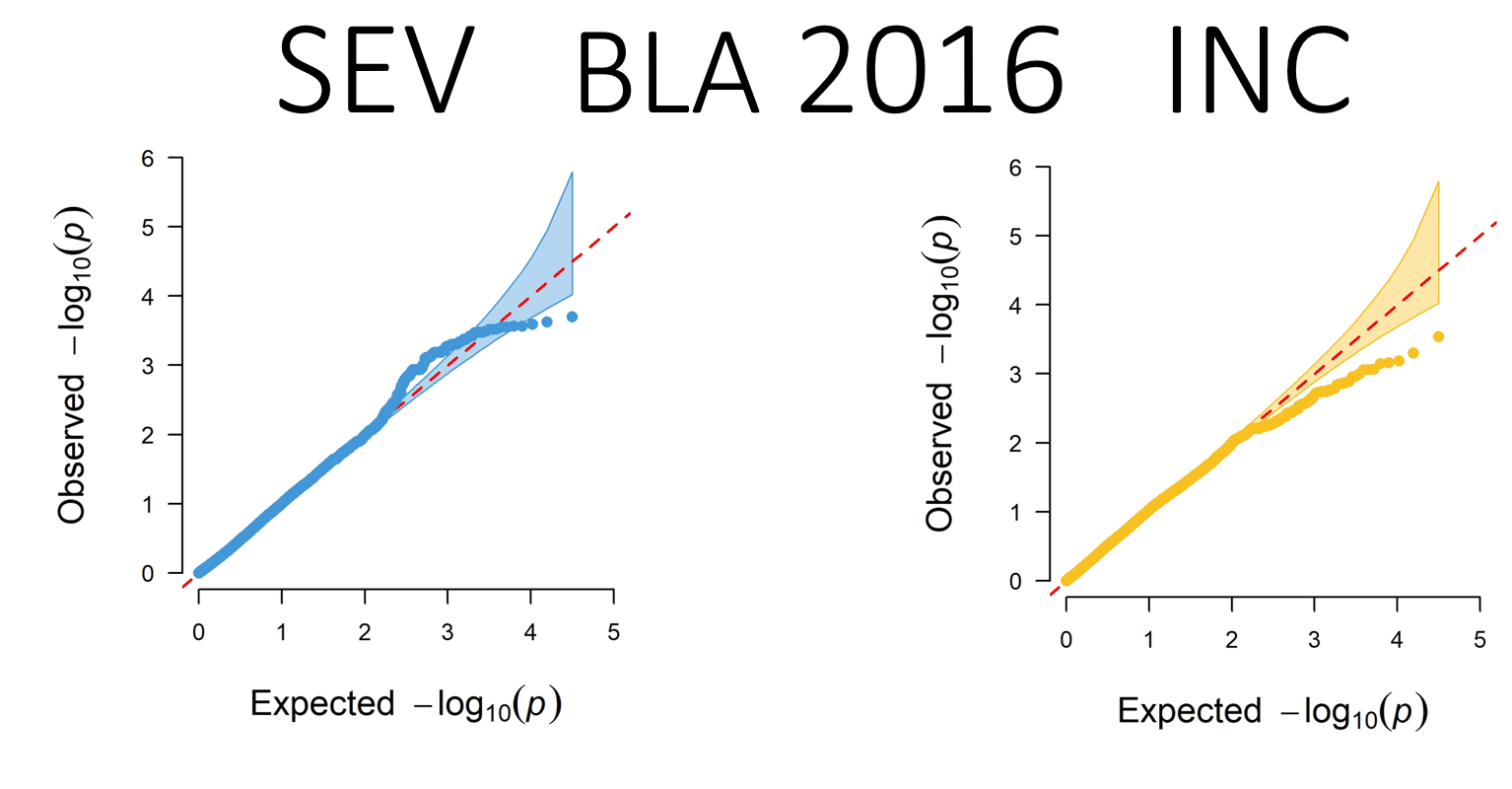 |
| --- |
| 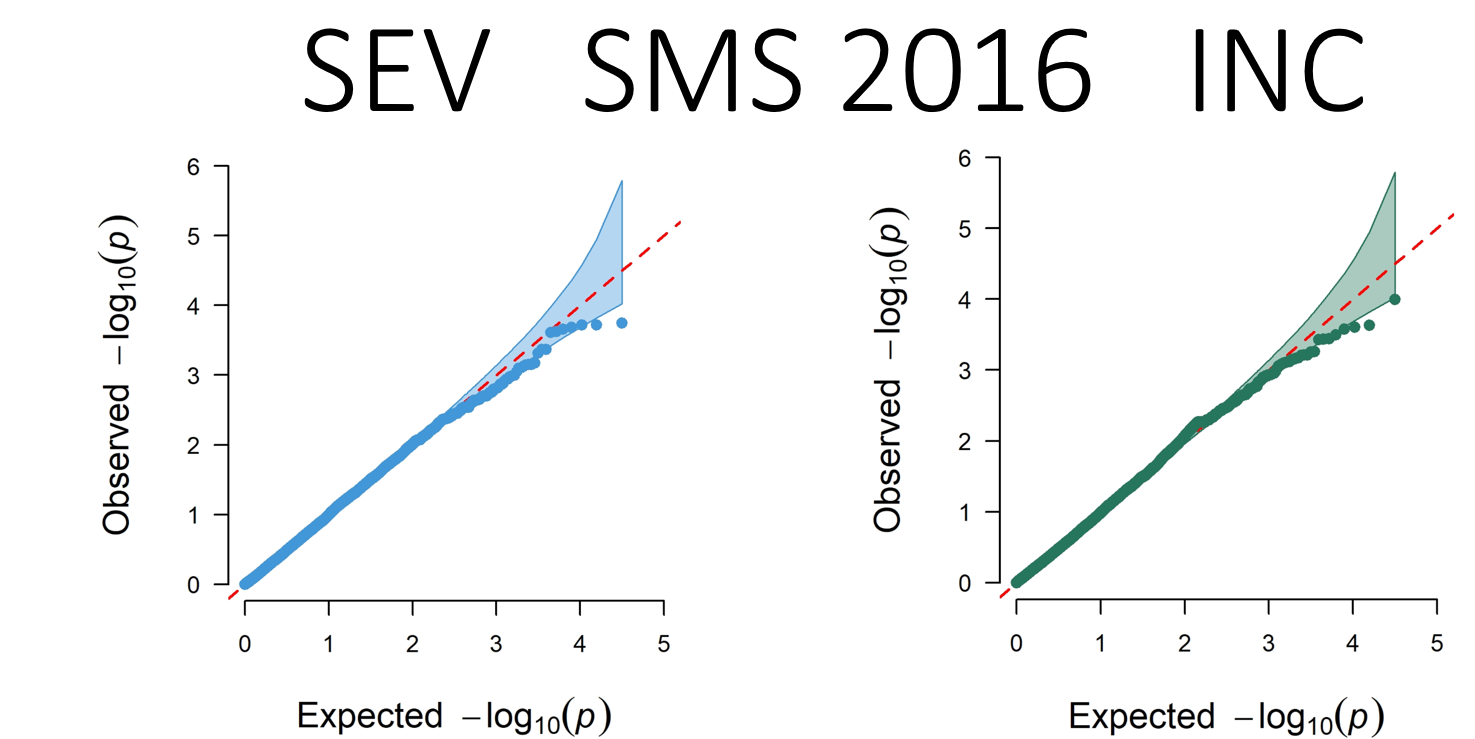 |

**Supplementary Figure 2** Quantile-Quantile (QQ) plots for disease severity (SEV), and incidence (INC) of soybean accessions evaluated for Cercospora leaf blight in Bossier City, LA (BLA) and Stoneville, MS (SMS) during 2016.

| 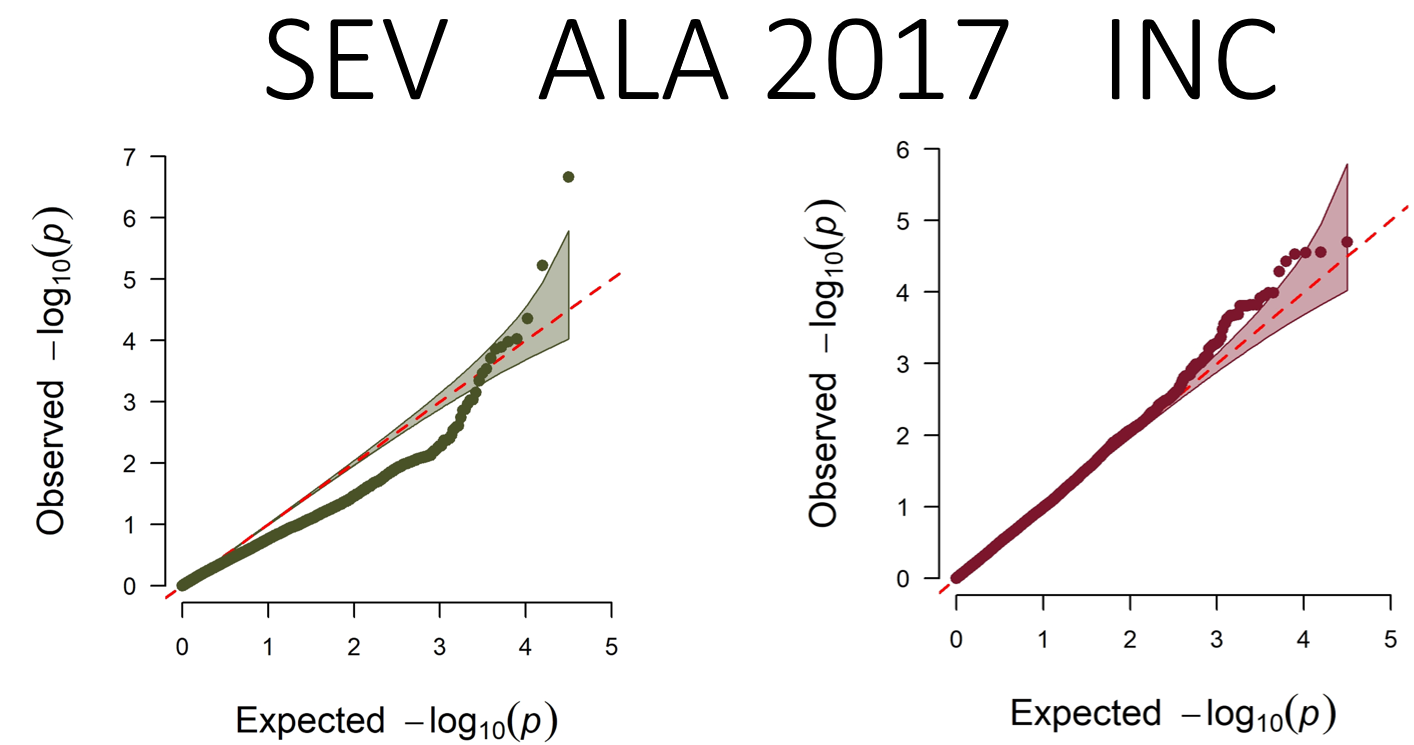 |
| --- |
| 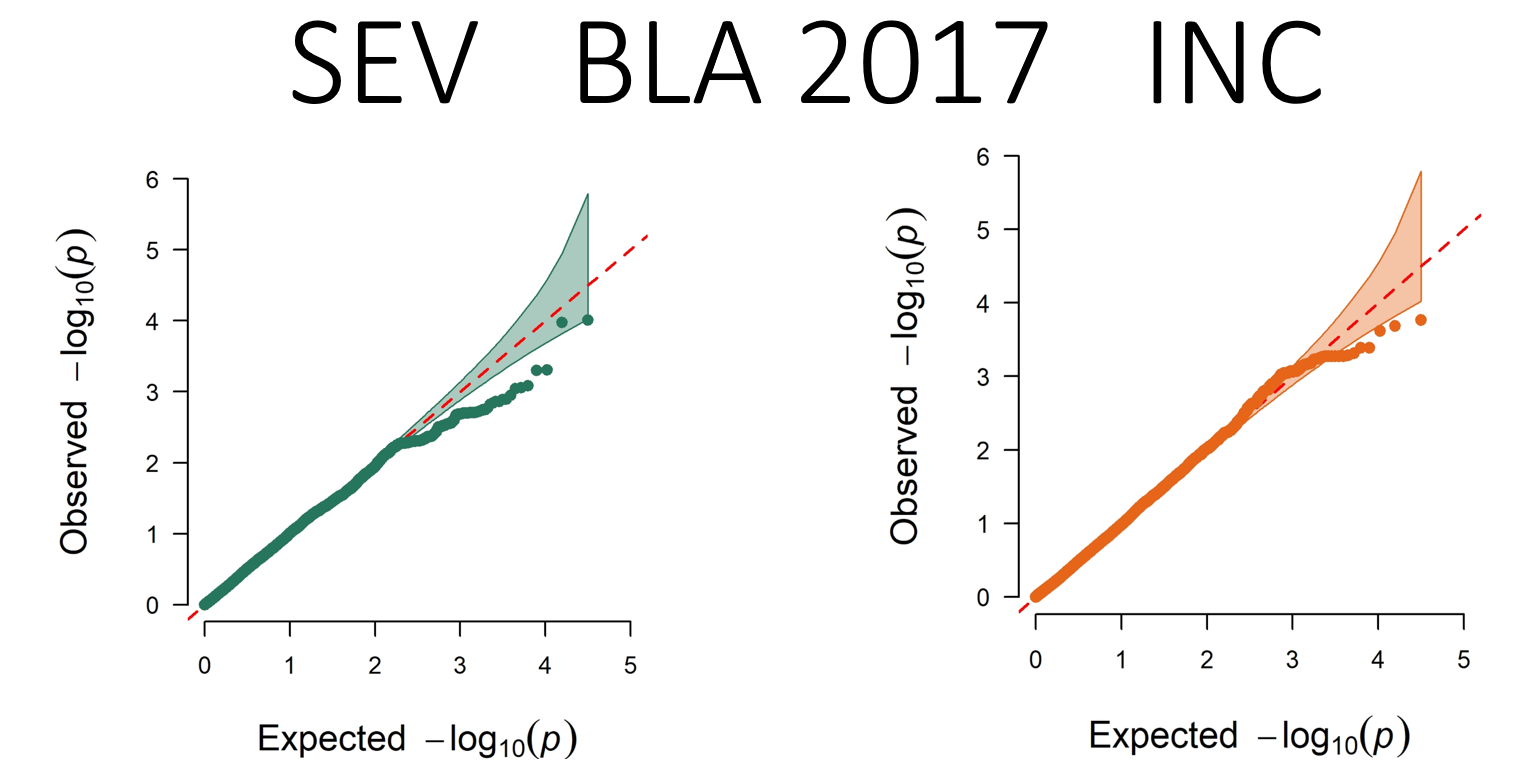 |
| 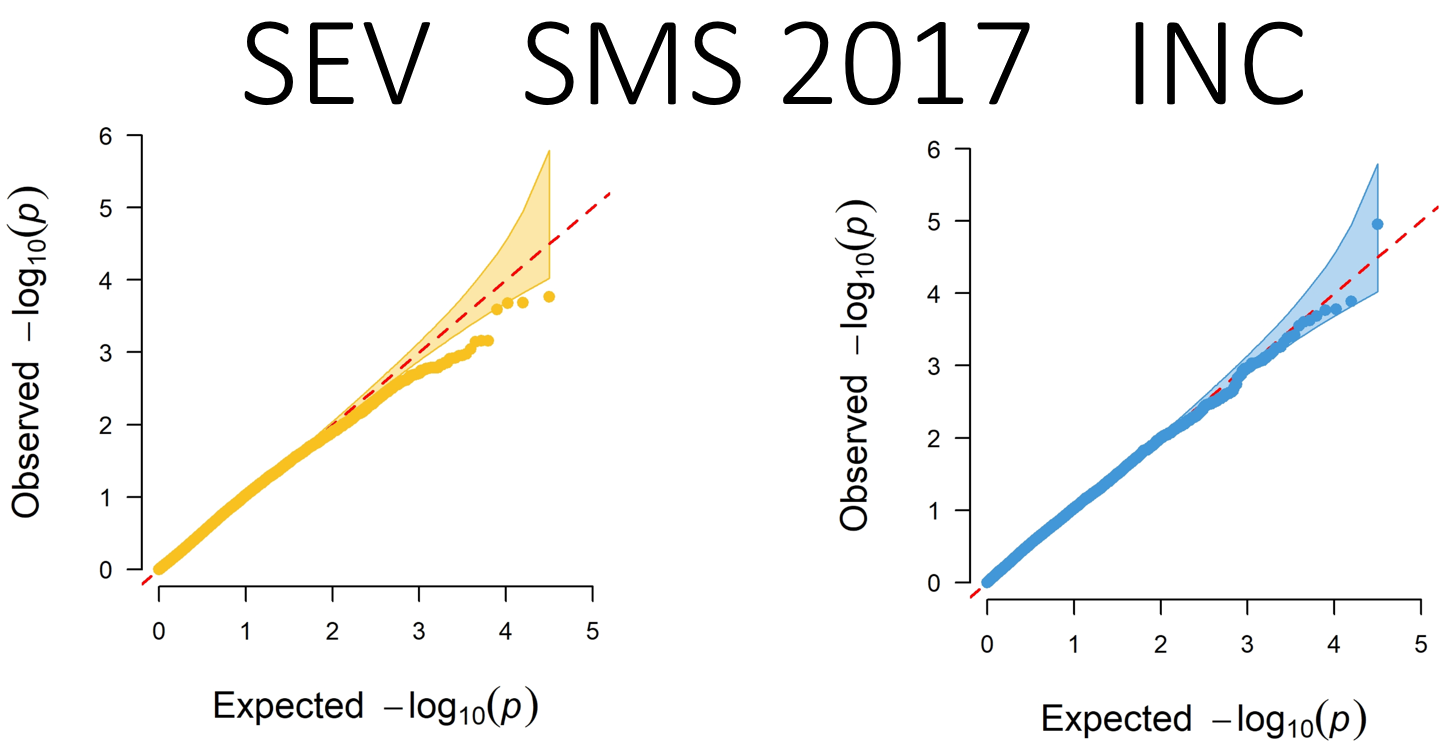 |

**Supplementary Figure 3** Quantile-Quantile (QQ) plots for disease severity showing the association between SNPs, disease severity (SEV), and incidence (INC) of soybean accessions evaluated for Cercospora leaf blight in Alexandria, LA (ALA), Bossier City, LA (BLA), and Stoneville, MS (SMS) during 2017.

| 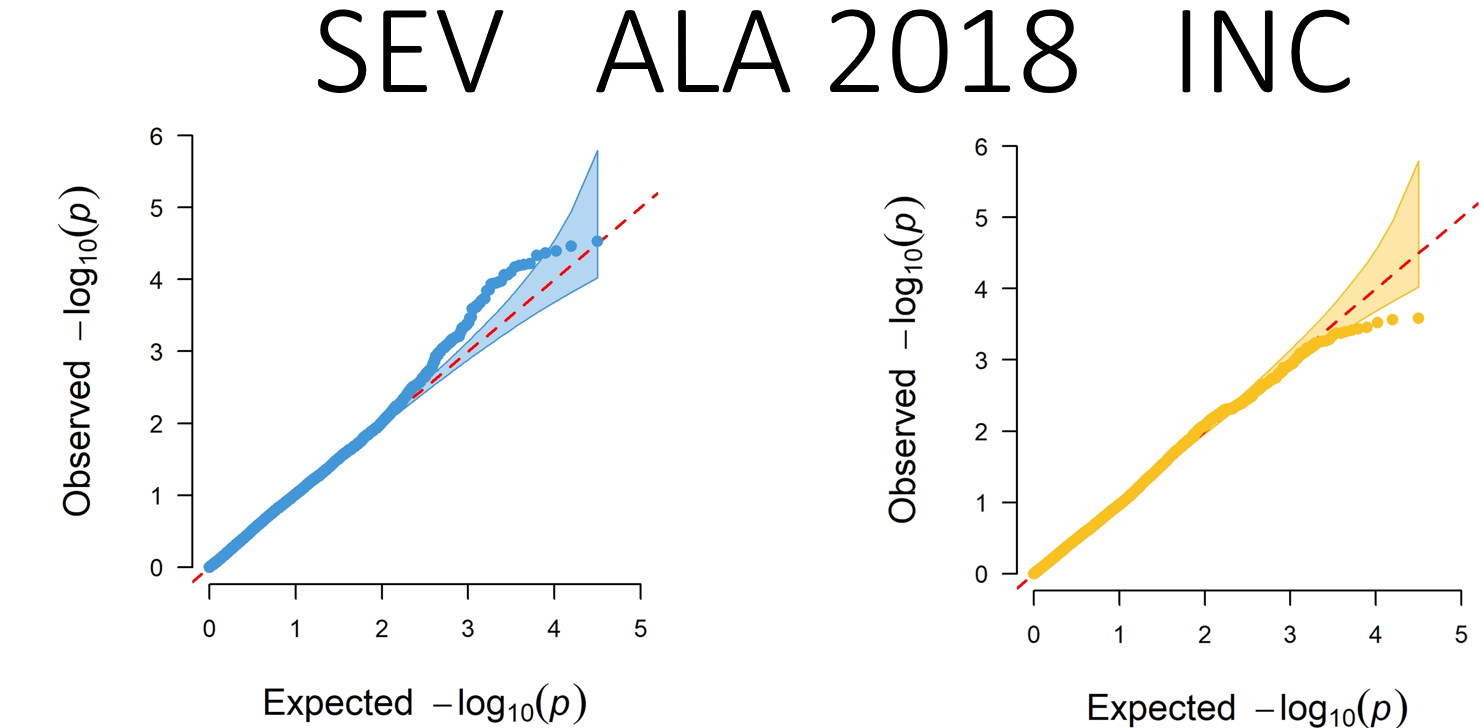 |
| --- |
| 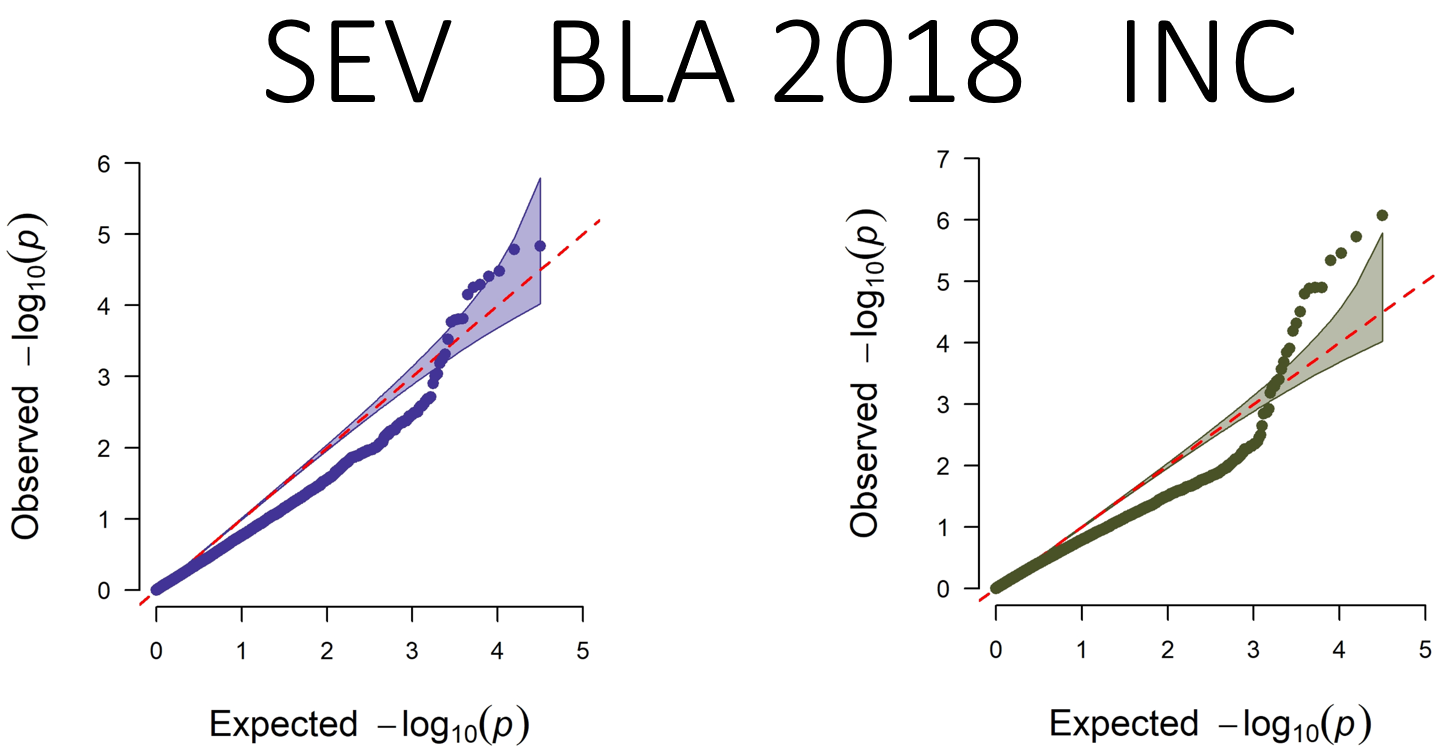 |
| 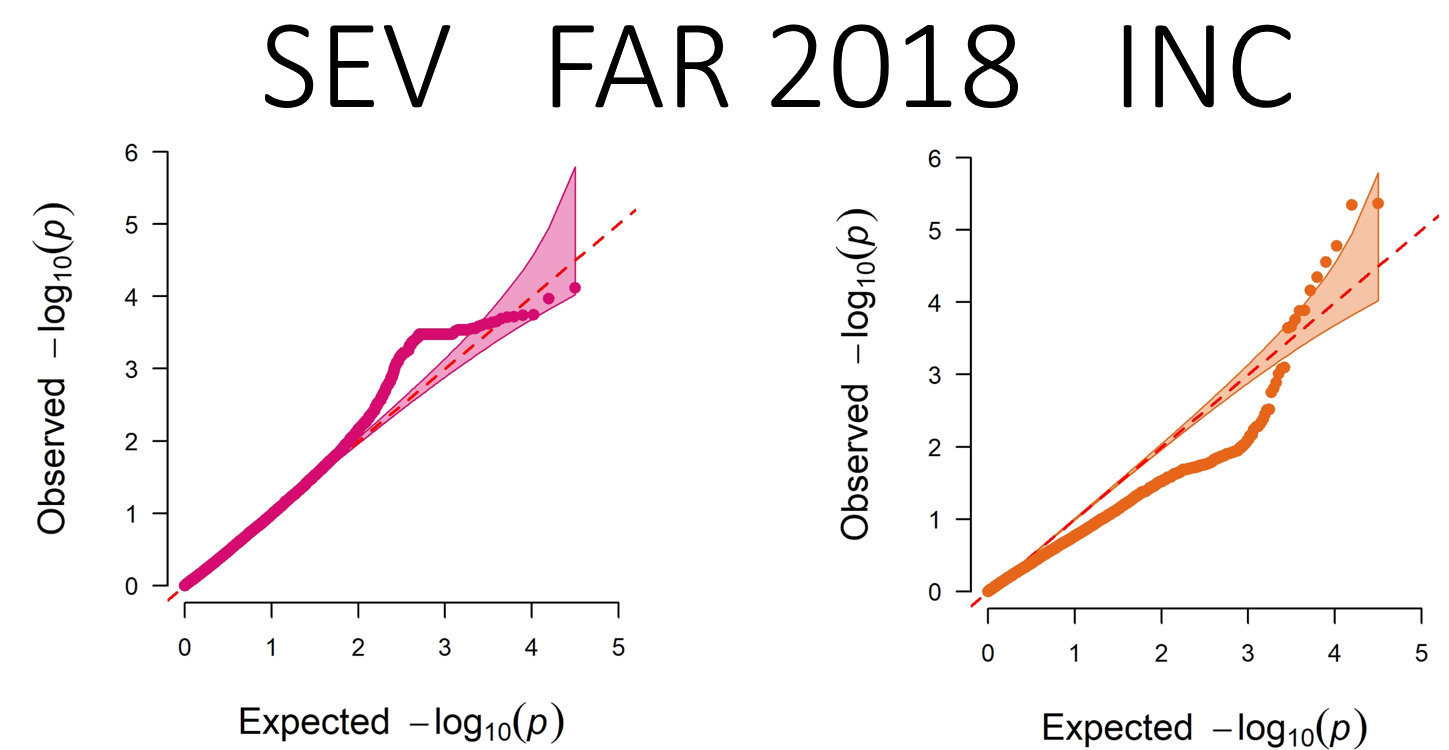 |
| 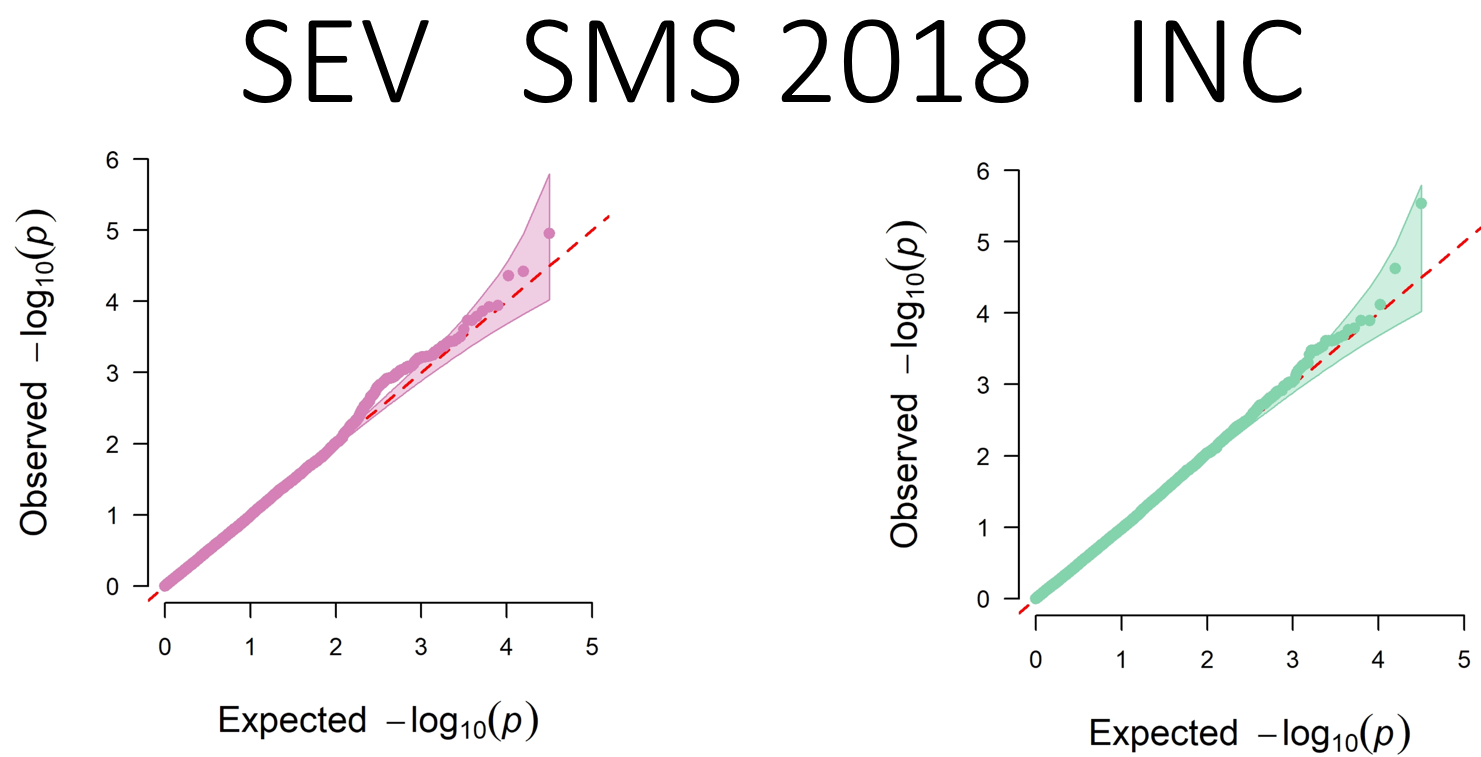 |

**Supplementary Figure 4** Quantile-Quantile (QQ) plots for disease severity (SEV), and incidence (INC) of soybean accessions evaluated for Cercospora leaf blight in Alexandria, LA (ALA), Bossier City, LA (BLA), Fayetteville, AR (FAR), and Stoneville, MS (SMS) during 2018.
